# Supplementary material for: Prevalence, Clinico-Bacteriological Profile, and Antibiotic Resistance of Symptomatic Urinary Tract Infections in Pregnant Women
Source: Antibiotics (Basel). 2022 Dec 25;12(1):33. doi: 10.3390/antibiotics12010033 (PMC9855124; doi:10.3390/antibiotics12010033)
Supplement: Supplementary file 1 [file antibiotics-12-00033-s001.zip › antibiotics-2116338-supplementary.pdf]

UTI Symptoms Questionnaire

1	In the past 7 days did you experience any one or more of the following symptoms?				
Please choose (√) more than 1 of the symptoms if applicable but 1 score for each of them if present		Did not have [0]	Mild [1]	Moderate [2]	Severe [3]
a.	Frequency of urination (going to the toilet very often)?	(4 times or less/day)	(5-6 times/ day)	(7-8 times/ day)	(9 times or more/ day)
b.	Urgency (A strong desire to pass urine that you cannot control)				
c.	Burning during the passing of urine				
d.	Pain during the passing of urine				
e.	Passage of blood in the urine				
f.	Feeling that the amount of urine is less and you have passed the urine incompletely				
g.	Pain or pressure in the lower part of your abdomen (tummy)				
h.	Pain in the lower back				
i.	-Fever with or without chills<br>-If fever, what is the measured body temperature	37.5^0 Celsius or less	$37.6\text{-}37.9^0$ Celsius	$38.0\text{-}38.9^0$ Celsius	39.0^0 Celsius or more
Go to question number 2 if you have any of the symptoms mentioned in question number 1					
2.	How bothersome was/were the symptoms for you over the last 24 hours?<br>[Please √ one of the below]				
a.	No discomfort/ No symptoms [0]				
b.	Feeling a little discomfort/ feeling a little worse than usual [1]				
c.	Feeling moderate discomfort/ feeling quite bad [2]				
d.	Feeling extreme discomfort/ feeling extremely bad [3]				
3.	Have you received any treatment for any of the symptoms so far?<br>If yes, what treatment have you received?				
4.	In this pregnancy, did you experience any of the symptoms before?<br>If yes, have you received any treatment for it, or has it resolved by itself?				
5.	Did you ever suffer from a urinary tract infection before this pregnancy?				
